# A Real-Time Health Monitoring System for Remote Cardiac Patients Using Smartphone and Wearable Sensors

**DOI:** 10.1155/2015/373474

**Published:** 2015-12-15

**Authors:** Priyanka Kakria, N. K. Tripathi, Peerapong Kitipawang

**Affiliations:** ^1^School of Engineering and Technology, Asian Institute of Technology, Phaholyothin Highway, Klong Luang, Pathumthani 12120, Thailand; ^2^International College of Medicine, Thammasat University, Rangsit Campus, Phaholyothin Highway, Klong Luang, Pathumthani 12120, Thailand

## Abstract

Online telemedicine systems are useful due to the possibility of timely and efficient healthcare services. These systems are based on advanced wireless and wearable sensor technologies. The rapid growth in technology has remarkably enhanced the scope of remote health monitoring systems. In this paper, a real-time heart monitoring system is developed considering the cost, ease of application, accuracy, and data security. The system is conceptualized to provide an interface between the doctor and the patients for two-way communication. The main purpose of this study is to facilitate the remote cardiac patients in getting latest healthcare services which might not be possible otherwise due to low doctor-to-patient ratio. The developed monitoring system is then evaluated for 40 individuals (aged between 18 and 66 years) using wearable sensors while holding an Android device (i.e., smartphone under supervision of the experts). The performance analysis shows that the proposed system is reliable and helpful due to high speed. The analyses showed that the proposed system is convenient and reliable and ensures data security at low cost. In addition, the developed system is equipped to generate warning messages to the doctor and patient under critical circumstances.

## 1. Introduction

During the recent decade, rapid advancements in healthcare services and low cost wireless communication have greatly assisted in coping with the problem of fewer medical facilities. The integration of mobile communications with wearable sensors has facilitated the shift of healthcare services from clinic-centric to patient-centric and is termed as “Telemedicine” in the literature [1]. In the larger perspective, telemedicine can be of two types: (1) live communication type, where the presence of the doctor and patient is necessary with additional requirements of high bandwidth and good data speed, and (2) store and forward type, which requires acquisition of medical parameters such as vital signs, images, videos, and transmission of patients data to concerned specialist in hospital [2, 3].

According to existing medical surveys, telemedicine has been adopted to take care of the patients with cardiac diseases, diabetes, hypotension, hypertension, hyperthermia, and hypothermia [4–8]. The most promising application is in real-time monitoring of chronic illnesses such as cardiopulmonary disease, asthma, and heart failure in patients located far from the medical care facilities through wireless monitoring systems [9]. Heart diseases have become one of the leading causes of human fatalities around the world; for instance, approximately 2.8 million people die each year as a result of being overweight or obese as obesity can lead to adverse metabolic effects on blood pressure and cholesterol which ultimately increases the risks of coronary heart disease, ischemic stroke, diabetes mellitus, and a number of common cancers [10]. According to WHO, it has been estimated that heart disease rate might increase to 23.3% worldwide by the year 2030 [11]. The treatment of such chronic diseases requires continuous and long term monitoring to control threat.

The ubiquitous social connectivity can be used in telemedicine for remote monitoring and offsite diagnoses. It should be noted that over 94% of the world population, that is, 6.8 billion people, are the subscribers of cell phone and about 2.7 billion subscribers are using Internet [12]. Cell phone subscription is increasing rapidly and might reach the level of 8.5 billion by the end of 2016 with 70% of smartphone users from developing countries. In addition, smartphones technology comprises various services such as location tracking, short message service, and access of WLAN/GPRS/3G which provides ubiquitous connectivity. There are extensive studies on use of mobile phones in healthcare and clinical practices illustrating the use of inbuilt applications of smartphones like GPS and location enabled services which offer independent survival of old age patient with fragilities [13–16]. Existing studies have also highlighted the uses of inbuilt apps in continuous monitoring and maintaining individual records [17]; for instance, in [18] another study, the authors discussed the benefits of existing smartphone health apps considering their credibility for continuous data flow, feasibility, portability, and power consumption. Nevertheless, the discrepancies such as battery consumption, calibration, and generation of false alarms have challenged the capabilities of smartphone apps in the implementation of real-time health monitoring and diagnosis.

An alternative to inbuilt smartphone sensors is wearable sensors that have been used for continuous monitoring, storing, and sending medical data to healthcare givers over distance. Existing studies with wearable sensors offer monitoring in applications like physiological, biochemical, and motion sensing [19–21]. These sensors have been used in monitoring health indicators and body positions of the patients, as well as in keeping track of sports and other activities [22, 23]. These wearable sensors are becoming promising due to the fact that these sensors are low cost, easily available, user friendly, accurate, and reliable. Many studies explored the clinical applications of wearable sensors in cardiovascular, neurological, asthma, and hypertension diseases [24, 25]. For instance, [26] developed a system to monitor congestive heart failure in patients, comprising a biosensor in the form of a ring that monitors heart data. Similarly, systems have been designed to monitor respiratory diseases which record acoustic signals by placing a microphone on the neck of the patient while breathing [26]. The framework consisted of a band-pass filter to reduce noise and other distortions in signals which helped to achieve approximately 90% of measurement accuracy. The research work was then extended for detecting apneas using algorithms [27]. Wearable technology is also useful in solving the issues of monitoring in motion artifacts by using multiple sensors integrated on a single chip. Integration of different sensors on same platform (tight fitting in garment) in order to monitor respiratory diseases is another kind of application. These systems are found to be better than spirometry but still require advancements to minimize motion artifacts [28].

Further, although wearable technology has contributed heavily in the advancement of healthcare monitoring systems, concerns are there that may affect performance of the healthcare monitoring systems. These concerns include (1) failing to use real-time data in the monitoring systems during testing of application, (2) battery issues, (3) security and privacy of the data collected from patients, (4) requirement of medical professional's recommendations at each step of the development, (5) clinical validation or experts' acceptability, and (6) user friendliness for the patients and for healthcare professionals. The integration of wearable technologies with mobile networks can offer new potentials of rapid, reliable, and secure information transfer from patients to the doctors [21, 29, 30].

The current study addresses the issue of integrating a wearable sensor with mobile technology by developing a remote monitoring system for heart patients. In this study, we propose a location based real-time monitoring system comprising a wearable sensor, mobile application, and a web interface to overcome some of the issues, as mentioned in the literature. The wearable sensor has been used to generate patient's diagnostic information which is then transferred to a smartphone wirelessly via Bluetooth low energy technology. Further, the collected information on the smartphone is transferred to a web interface via Wi-Fi/3G. The proposed system has the ability to generate emergency alerts on the basis of predefined values by comparing patient's data to inform the doctor if there is a requirement of checkup or investigation. Furthermore, various types of sensors have been used and results are compared to identify the most promising sensor providing most accurate results close to the conventional systems. The developed system has been evaluated under the supervision of the experts.

## 2. Materials and Methods

This study develops a remote monitoring diagnostic framework to detect underlying heart conditions in real-time which helps avoiding potential heart diseases and rehabilitation of the patients recovering from cardiac diseases. The proposed real-time monitoring system is compatible to use various wearable sensors to extract medical information which helps finding out multiple parameters such as heart rate, blood pressure, and body and skin temperature at the same time. These cardiac parameters help early detection of diseases such as arrhythmia, hypotension, hypertension, and hyperthermia through alarming system based on upper and lower threshold values. Similar to the existing monitoring systems, the developed system has two interfaces, one for patients and other for the doctor. The patient interface is comprised of wearable sensors which extract medical information of the patients and transmit to an Android based listening port via Bluetooth low energy. The listening port transfers this information to web server which processes data to show reports on doctor interface. The details of the system architecture, components, data processing, and alarming system are explained as follows.

### 2.1. System Architecture

**Figure 1:**
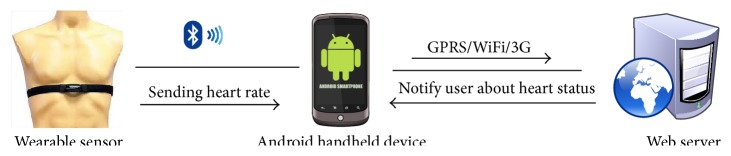
System architecture for remote patient monitoring system.

The system architect is three-tier comprising (1) a patient interface, that is, wearable biosensors' tier, (2) Android handheld device, that is, a smartphone, and (3) a web portal as shown in Figure 1.

The first tier of the system is patient's interface which consists of multiple wearable sensors used to collect medical information of the patient. This tier transmits real-time data wirelessly from wearable devices worn by the patient to second tier of the system via Bluetooth low energy. The second tier consists of an Android smartphone used to extract patient's information from wearable sensors. Android smartphone with inbuilt wireless networking has the ability to communicate with web portal via GPRS, 3G, or other Wi-Fi networks. In addition, the inbuilt GPS application of the smartphone helps to find out the location of the individuals under observation. In third tier, web portal extracts data from SQLite internal data base and transfers data to online MySQL via GPRS/3G or Wi-Fi. Web portal is a platform that acquires the data of multiple patients wearing wearable sensors and displays them on web interface, also known as doctor's interface, along with location and personal information for identification. In this study, three types of wearable sensors are used to extract heart rate, blood pressure, and body temperature information of the patients.

The proposed system has the ability to use multiple sensors which enables simultaneous monitoring of several heart parameters from multiple patients. The facility of using multiple sensors at same time to obtain required data increases applicability of the developed system and assists in comparing the accuracy of various sensors. The focus of this study is to develop a real-time diagnostic system for remotely located heart prone patients by measuring heart rate, blood pressure, and body temperature using wearable sensors.

Heart rate or heart pulse is a palpable rhythmic expansion of an artery produced by electric flow of blood and recorded as number of pulses in one minute [31]. The change in pulse rate can be due to either body activity or any medical disorder. The effect of age on pulse rate considered as normal range is different for different age groups. The importance of measuring the accurate heart rate is understood and should not be ignored. The measuring accuracy of the sensor has direct impact on accuracy of the heart rate measurement in real-time monitoring systems. Therefore, the selection of an accurate heart rate monitoring device is of prime importance in early detection of underlying heart diseases.

Zephyr BT is a wearable sensor which has been used to extract heart rate information of the patients requiring continuous monitoring. The selection of Zephyr BT sensor is made on the basis of accuracy, reliability, cost, availability, and comfort. Bluetooth low energy technology makes this sensor feasible to use for longer time, which ultimately helps in increasing the system's durability and minimizes the risk of false alarms. Furthermore, Zephyr BT is compatible with Android version 4.4 and above.

The second measuring parameter of our monitoring system is blood pressure (BP). Blood pressure refers to the force exerted by the flow of blood against the atrial wall during heart contraction (systole) and heart expansion (diastole). An acute trend in BP (blood pressure) is considered as early indication which often helps medical experts diagnosing serious patients. Omron Wireless Upper Arm blood pressure monitor is used for extraction and wireless transmission of blood pressure information to the server. The Omron Wireless Upper Arm blood pressure monitor has been chosen due to its easy handling, light weight, and long term battery operating features. Furthermore, it has the ability to record 200 previous values of BP along with transmission to handheld listening port.

Body temperature of the patient is the third parameter under the scope of this study which is required to be measured for early detection and diagnosis purposes. G plus, Bluetooth based temperature sensor is used to monitor body temperature. The temperature information is then transmitted to handheld listening port using wireless communication.

### 2.2. Data Transmission from Wearable Sensors to Android Listening Port

**Figure 2:**
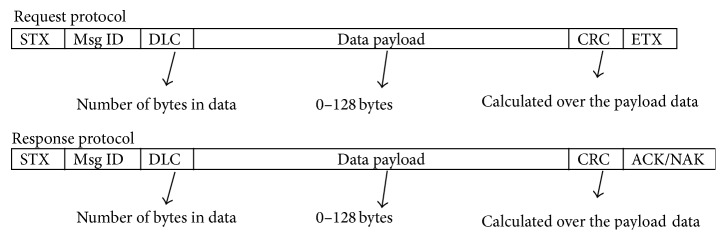
Message format for communication.

The data transmission process from sensor to Android listening port via Bluetooth is shown in Figure 2. STX denotes the start of text in ASCII character by delimiting the message. Message ID is in binary format which is used to identify the type of message. The standard message ID of Zephyr BT for heart monitoring is 0 × 26. Number of bytes within the data payload has been specified by data length code (DLC). Data payload contains the actual data transferred from Zephyr BT to listening port of the smartphone comprising data ranges from 0 to 128 bytes. CRC is an 8-bit CRC polynomial of 0 × 8C (CRC-8). The accumulator is being initialized to zero before starting the CRC calculation. ETX is a standard ASCII control character that ends message. Bluetooth technology is a short wavelength radio transmission in ISM band from 2400 to 2480 MHz used for standard data transmission in short ranges. In current study, this technology is used as a serial port transmission having sufficient baud rate of 115200 bps.

### 2.3. Creating Interphase for Android Listening Port

The listening port application has been developed in Android studio interface. The developed listening port is installed in an Acer E3 smartphone which acts as an Android handheld listening port of the system to receive information from first tier of the system via BLE (Bluetooth low energy). Once the connection is established, the listening port of the system starts scanning process via* startLeScan()* to detect BLE sensor and it stops immediately as soon as the detection process is complete (connection is created using* connectGatt()* approach). Heart rate data collected on listening port of the system using GATT attributes is in hexadecimal code which is then converted to integer value to determine average heart rate. Once data is collected from the sensor,* close()* function closes the listening port application. Heart data from smartphone is then transferred to data processing server using extensive messaging and presence of protocol (XMPP) networks. For data security, the smartphone keeps history of the records in SD card memory. The data preprocessing server processes data to compute average heart rate with respect to time. Furthermore, data preprocessing server generates alarms in abnormal conditions to notify the doctor. In addition, inbuilt GPS in smartphone helps locating the geographic location of the patient.

**Figure 3:**
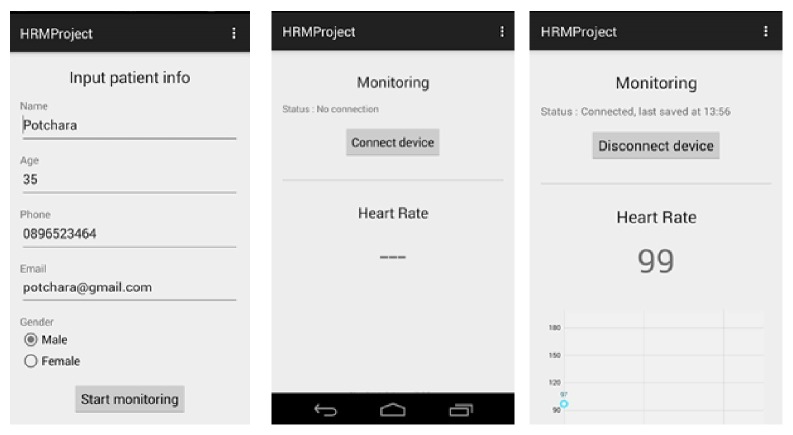
Application interface of listening port: patient basic information, connection notification, and real-time monitoring.

The snapshots of listening port interface are shown in Figure 3. First interface of the listening port is a registration form which is designed to collect personal information of the patient such as name, age, gender, and contact details. Second interface shows the connectivity status between the sensor and listening port. In third interface, real-time heart rate data stream appears with respect to time.

### 2.4. Web Interface

**Figure 4:**
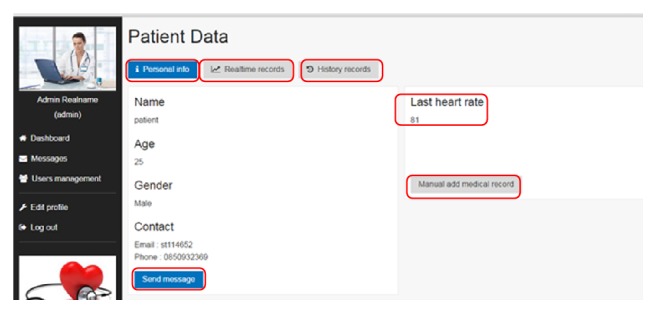
Patient data interface in web application.

Web interface enables several physicians, doctors, and medical centers to view and diagnose patients' medical status simultaneously. However, to ensure data visibility only to authorized doctor/physician, web portal requires user ID and password. Web interface is implemented using Larval PHP framework. The data from the listening port is presented to the doctor on web portal in order to check medical status of the patient. Web portal is an interface between doctor and patient as shown in Figure 4.

#### 2.4.1. Modules of Web Interface

*Patient Data*. This module consists of the patient's personal and medical records. Real-time data acquired by wearable sensors has been shown with respect to time. It contains the medical history of individual patient after getting registered at Android listening port device.

**Figure 5:**
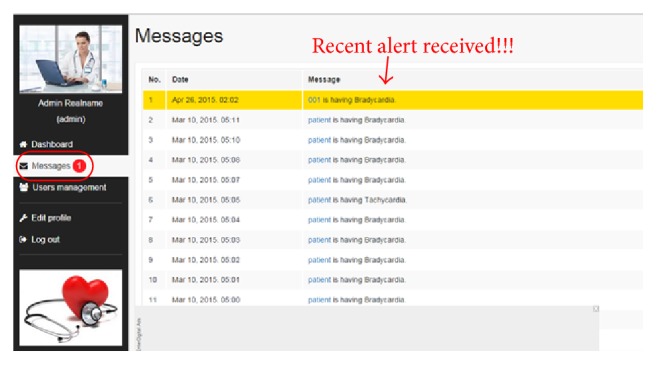
Alarming interface of remote patient monitoring system.

*Alarming Messages*. This module contains alarming messages generated at Android handheld listening port. Extracted physiological parameters give the alarming signals after comparison with assigned threshold values. These alarming signals indicate abnormalities like arrhythmia, hypotension, hypertension, fever, and hypothermia. Some real-time alarms from patients are shown in Figure 5.

*User Management*. User management allows the involvement of multiple doctors in the web application for diagnosing patients. In case of large data from multiple patients, several doctors can get involved in monitoring and diagnosing processes. User management module can contain 100 users including doctors, patients, and nursing staffs for keeping record of patients.

**Figure 6:**
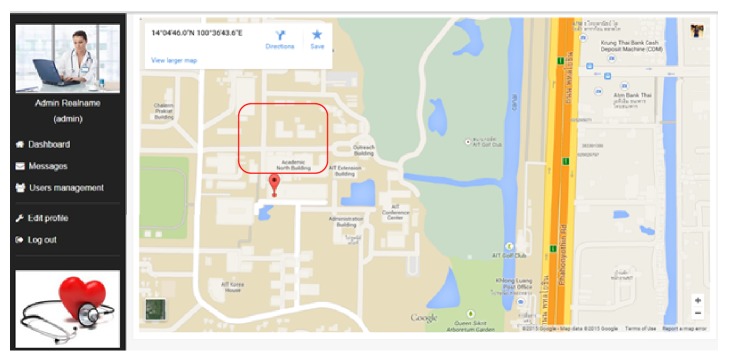
Location record of remote patient monitoring system.

*Location Records*. The location records of patients are shown in Figure 6. After knowing about the abnormalities, the doctor can immediately track the current location of the patientwhich helps in both (1) reaching the patient and/or (2) sending ambulance to transfer the patient to hospital in case of serious emergency.

**Figure 7:**
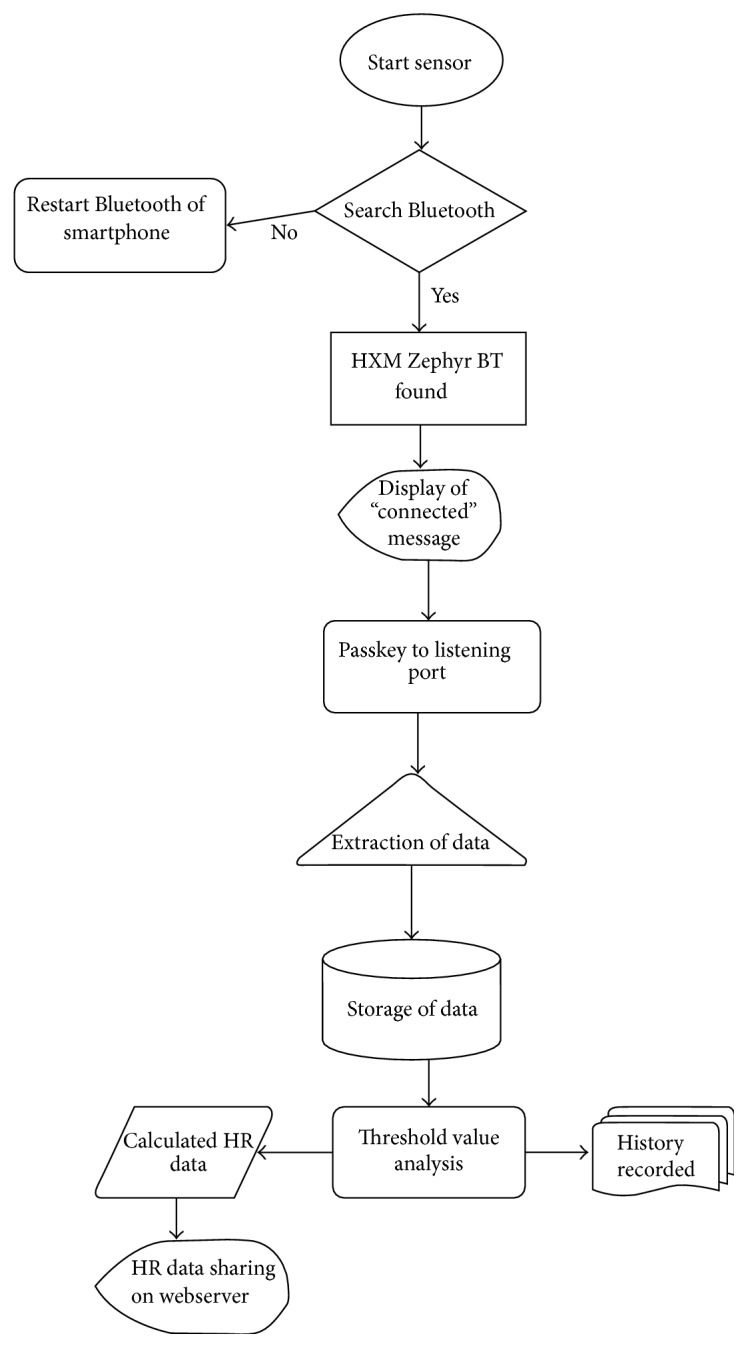
Flowchart of the remote patient monitoring system.

Figure 7 depicts data communication extracted by Zephyr BT (heart rate sensor) from patient to the doctor. For monitoring heart rate, patient needs to wear the sensors and once the connection is established, the sensors will start transmitting heart information of the patient to the listening port of the smartphone every 1.008 seconds at frequency of 1 Hz without requiring acknowledgements. A passkey has also been assigned for secured data acquisition from sensors to the listening port of the smartphone. From the Android listening port, the heart rate data is transferred to the web application.

### 2.5. Adaptive Alarming System

**Figure 8:**
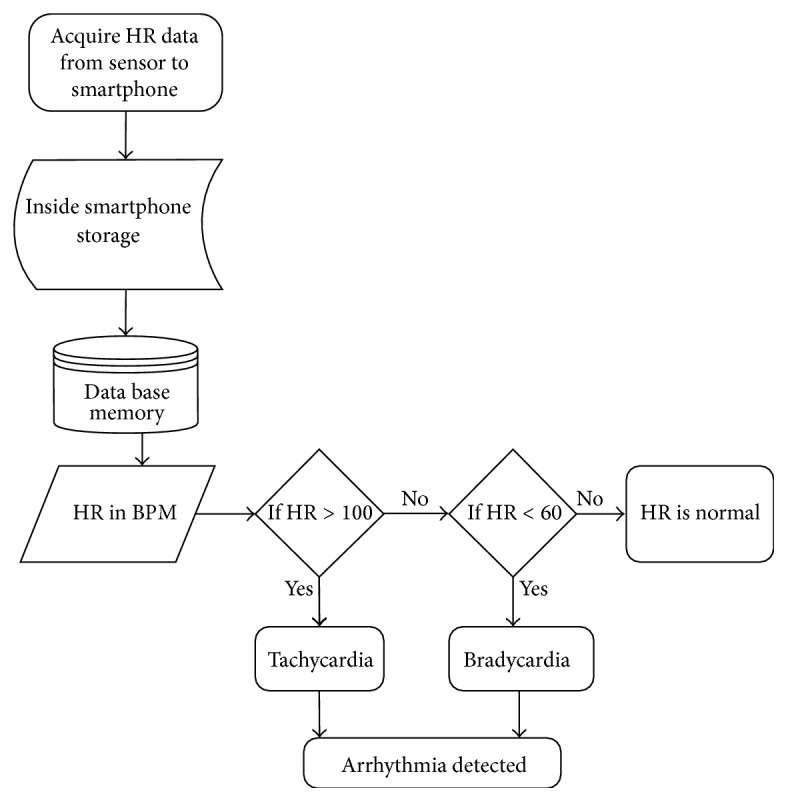
Flowchart of alarming mechanism.

The proposed system contains adaptive alarming system which generates alarms to notify the concerned doctor in case of emergency. The alarming system is based on threshold algorithm as shown in Figure 8. The threshold values have been taken from the report of American Heart Association (AHA) [32] and European Society of Hypertension [33] and also after discussing with cardiologists, as shown in Table 1. The predefined handler task generates alarm if average heart data deviates from the threshold value. The alarming system also generates a message to notify the user about the abnormality.

Alarming mechanism basically consists of data visualization, statistical preprocessing, and notifications. These three components come under JAVA classes providing scalability application using polymorphism. Decisions in alarming mechanism are based on mathematical model [34], which describes the cardiovascular system during various activities.

The proposed alarming system is a generalized monitoring model that works on the principle of upper and lower threshold values. The threshold values on the basis of different age groups are presented in Table 2. The proposed alarming mechanism can be customized for individual monitoring due to the fact that the proposed alarming system accepts various threshold values, and values of the threshold levels can be defined on the basis of cardiac vital parameters of the individuals. The customized monitoring helps in setting adaptive boundary limits which keep on changing throughout the monitoring phase. The proposed system compares heart rate after every ten minutes with previous values to find out if any change occurred.

Robust scoring mechanism has been applied in alarming module. In this module, the information of heart rate, blood pressure, and body temperature is collected through sensors which is then transmitted and collected on a platform called handheld listening port. Fuzzy logic approach is used to interpret the preprocessed data in association with diagnosis concepts such as bradycardia, tachycardia, hypertension, hypotension, fever, and hypothermia. Different threshold values have been derived using normal and abnormal vital sign ranges annotated by a physician as well as from the literature [35]. The alarming system works as shown in Box 1.

## 3. Result and Discussions

In this study, a real-time heart monitoring system is developed for chronic disease management. Various cardiac parameters such as heart rate, blood pressure, and temperature are being acquired using wearable sensors. Android listening port has been created to receive and store medical information of the patient which is then transmitted to the web interface using wireless communication. Web interface has been designed to be on the doctors' side so as to inform them of the medical status along with location of the patient in real-time. The accumulated data is stored in data server which pushes the information to doctor's web interface and ultimately, data of the patient located in remote area is made visible to the doctor sitting in super specialty hospital.

In this section, the practical implementation of the proposed real-time monitoring system is examined to determine the ability, accuracy, and performance of the system. The practical implementation process is comprised of real-time implementation of the system to heart prone patients in hospitals.

Prior to the integration of wearable sensors into the system, calibration of the wearable sensors has been done with respective conventional measuring devices, used in hospitals. In detail, Bluetooth based blood pressure sensor is evaluated with manual mercury column meter which shows that blood pressure sensor is more accurate on left-hand side. Heart rate sensors are tested with conventional machine and also with one-minute manual reading and results obtained are found to be accurate. Similarly, temperature sensor is calibrated with mercury thermometer and found to be accurate.

The practical implementation process of the system involves the participation of forty heart patients. Each patient was fitted with various wearable sensors to obtain medical information. Each subject was tested with respective conventional machines for evaluation. The testing protocol directed for each subject continued for a 10-minute acclimation period. Two data sets (wearable sensor and conventional devices) were assumed to have normal distribution and the resulting *P* value was 0.0003 (paired *t*-test). Statistical information of patients has been given in Tables 3 and 4.

The proposed system is designed to send alarm message immediately after getting arrhythmia. The system has the ability to notify the conditions of tachycardia and bradycardia. The experimental validation process has been done for remote patients residing 10–100 km away from the hospital (server).

### 3.1. Alarming Response Time

The alarming system compares medical information of the patient with given threshold values and generates an alarming message if the measured value crosses upper or lower threshold limits. Typical 3G DTAC network has been used on Acer E3 (Liquid Model). Moreover, the performance of the system has been checked for both Wi-Fi and 3G communication protocols. It is found that the time taken for sending an alarm message from patient to doctor is 30 seconds with Wi-Fi networks, while in case of 3G networks, the time taken by an alarm message from patient's interface to the doctor is 56 seconds. The average transmission time of an alarming message for Wi-Fi and 3G network is shown in Table 5.

According to American Heart Association (AHA), 90% of the patients cannot recognize themselves as being at high risk before cardiac attack. Hence, the risk can be minimized by giving them medically calibrated wearable sensors with alarms in case of abnormality [36]. AHA recommended that ideal time in between sudden fall or rise in cardiac vital signs and sending an alarm message to cardiologist is in between 4 to 6 minutes which is called the golden period of saving a heart patient. The time required for an alarm message in proposed monitoring system under both wireless protocols is less than the golden period. Even in outskirt areas with poor network, the data transmission rates are quite acceptable as per medical standards. Present study demonstrates the applicability of remote care monitoring and it assures 24/7 continuous screening of patients residing at a considerable distance from super specialty hospitals.

Some existing studies about Smart Vest [37] and LOBIN [38] share a similar approach to monitor remote patients using wearable textiles. Smart Vest uses wireless transmission from textile to a remote web server whereas LOBIN uses wireless transmission boards and distribution points in remote areas. Despite advantages of the systems, there are some concerns, like patient's comfort; they may find garments with wired sensors uncomfortable. By overcoming the concern, current system uses wearable sensors that are physically comfortable for wearing. Another important feature of the developed system is the use of 3G network which provides wide coverage area as compared to existing system. This wide area coverage allows free mobility of the patient which helps the patient to perform daily without disturbance while being monitored. In contrast, the systems proposed in the literature provided limited coverage area. For instance, in [39] authors designed a multihoping network for multipatient remote monitoring system that offered only 10-meter coverage area.

From the experiments, it has been found that one physician can monitor 15–25 patients if solely appointed on proposed system; however, this number reduces to 5–12 patients if a physician is requested to monitor patients through proposed system along with regular work in hospital. Furthermore, it is important to mention here that the number of patients depends on the condition of the patients in hospital as well as patients monitored by the proposed system.

## 4. Conclusion

Increasing rate of chronic diseases in aging population is becoming a serious concern due to lack of sufficient facilities and extremely high cost. The situation is even worse for the people residing in remote areas far from medical facilities as delay in diagnosis and treatment may lead to death. Timely diagnosis and treatment can solve these issues to a great extent. The advancements in wireless communications and wearable sensor technology open up the opportunity of real-time healthcare monitoring systems. In this study a real-time heart monitoring system for heart patients located in remote areas has been proposed. The developed system is comprised of wearable sensors, Android handheld device, and web interface. The system is adaptable and has the ability to extract several cardiac parameters such as heart rate, blood pressure, and temperature of multiple patients simultaneously. The extracted data is being transmitted to Android handheld device using Bluetooth low energy which is then transmitted to web application for further processing. Web application processes received data to show medical status of the patient along with personal information such as age, gender, address, and location on web interface. An alarming system based on threshold values has also been designed which sends alert message to the doctor in case of abnormalities such as arrhythmia, hypotension, hypertension, fever, and hypothermia.

In order to evaluate and show the practical implementation, the developed system has been used to monitor forty (40) cardiac patients located far away from the designed web application. The data obtained through the developed system is found to be significantly acceptable. Furthermore, for remote monitoring validation the system is tested under Wi-Fi and 3G wireless protocols to find out about time delay, that is, the time taken to send data from patient's interface to doctor's interface, and found that the message sending time for both wireless protocols is under acceptable range of medical standards (4 to 6 minutes as per American Heart Association).

The scope of this study is the development and implementation of real-time monitoring system for remote patients using wireless technology. The developed system would inform the doctor in case of emergency through alarms; however, delay in alarms might occur due to weak signals of 3G networks in some remote areas. Though the delayed alarming time is still within the golden period of time it should be considered in future research. As wireless technology is emerging day by day, the use of latest wireless technology may overcome these issues which ultimately increases the applicability and usefulness of the proposed remote monitoring system. Furthermore, false alarms can be generated due to the battery issues of sensors and smartphone. The research can be extended to overcome these battery and false alarm limitations.

## Figures and Tables

**Box 1:**
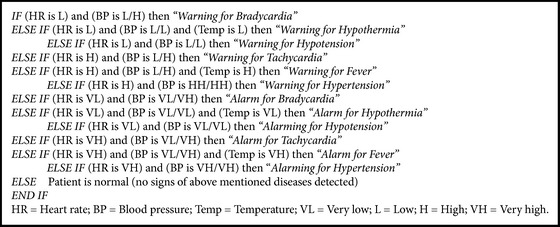


**Table 1:** Threshold values for alarming mechanism.

Sinus rhythm type	Threshold value of heart rate, blood pressure, and temperature

Normal	60 ≤ HR ≤ 100 (beats/minute), BP = 100–140/60–80 mmHg, and temperature = 36.5–37.5°C
Bradycardia	HR ≤ 60 (beats/minute)
Tachycardia	HR ≥ 100 (beats/minute)
Hypertension (Stage 1)	Blood pressure = Sys/Dys ≥ 140/90 mmHg
Hypertension (Stage 2)	Blood pressure = Sys/Dys ≥ 150/95 mmHg
Hypotension	Blood pressure = Sys/Dys ≤ 90/60 mmHg
Fever	Temperature ≥ 37.8°C
Hypothermia	Temperature ≤ 35.0°C

**Table 2:** Age-wise threshold values for adaptive alarming mechanism.

Parameters/age	18–35	36–64	Above 64

Normal heart rate	72–75 (BPM)	76–79 (BPM)	70–73 (BPM)
Bradycardia	HR ≤ 55	HR ≤ 60	HR ≤ 65
Tachycardia	HR ≥ 110	HR ≥ 120	HR ≥ 100
Hypertension	BP ≥ 150/100	BP ≥ 145/95	BP ≥ 140/90
Hypotension	Systolic BP < 85 mmHg	Systolic BP < 96 mmHg	Systolic BP < 117 mmHg
Fever	Temperature ≥ 37.2°C	Temperature ≥ 37.5°C	Temperature ≥ 36.9°C
Hypothermia	Temperature < 35.5°C	Temperature < 35.1°C	Temperature < 35.0°C

BPM represents beats per minute.

**Table 3:** Mean value of patient data.

S. number	Parameter	Mean values
1	Number of patients	40
2	Age	41
3	Gender (M/F)%	36/64
4	Heart rate	79 BPM
5	Blood pressure	123/71
6	Temperature	36.5°C

**Table 4:** Statistical data of patients.

S. number	Parameter	Age	Heart rate	Blood pressure	Temperature
1	Minimum	25	62	67/45	32.5
2	Maximum	66	120	190/106	36.6
3	Range	41	93	123/61	4.1
4	Standard deviation	13.5	16.5	21.5/13/5	0.3
5	Median	37	96.5	126/68	36.5
6	Mode	49	84	124/68	36.6

**Table 5:** Average data transmission time using Wi-Fi and 3G.

Alarm for	Average time b/w sending and receiving alarm in Wi-Fi (H:M:S)	Average time b/w sending and receiving alarm in 3G network (H:M:S)
Tachycardia	00:00:29	00:00:58
Bradycardia	00:00:30	00:00:59
Hypertension	00:00:31	00:00:51
Hypotension	00:00:33	00:00:57

Note: the value for each type of alarm is the average value of 20 alarms.
